# Factors Associated With the Microbiome in Moderate–Late Preterm Babies: A Cohort Study From the DIAMOND Randomized Controlled Trial

**DOI:** 10.3389/fcimb.2021.595323

**Published:** 2021-03-01

**Authors:** Clara Yieh Lin Chong, Tommi Vatanen, Tanith Alexander, Frank H. Bloomfield, Justin M. O’Sullivan

**Affiliations:** ^1^ Liggins Institute, The University of Auckland, Auckland, New Zealand; ^2^ Infectious Disease & Microbiome Program, The Broad Institute of MIT and Harvard, Cambridge, MA, United States; ^3^ Neonatal Unit, Kidz First, Middlemore Hospital, Auckland, New Zealand; ^4^ The Maurice Wilkins Centre, The University of Auckland, Auckland, New Zealand

**Keywords:** moderate–late preterm infant, socioeconomic status, ethnicity, gut microbiome, early life nutrition

## Abstract

The gut microbiota of preterm infants is affected by perinatal factors and, in turn, may impact upon infant health. In this study, we collected fecal samples at Day-10 (D10) and 4-months corrected-age (4M) from 227 moderate–late preterm (MLPT) babies enrolled in a randomized controlled trial of nutritional management. A total of 320 samples underwent 16S amplicon sequencing, and shotgun metagenomic sequencing was performed on 94 samples from the 4M time point. The microbiome of babies whose families lived in lower socioeconomic status (SES) areas exhibited a significantly higher microbial alpha diversity at D10 (Wilcoxon test*, p* = 0.021), greater abundance of *Bifidobacterium* (linear model, q = 0.020) at D10 and *Megasphaera* (q = 0.031) at 4M. Hospital of birth explained 5.2% of the observed variance in 4M samples (PERMANOVA, *p* = 0.038), with *Staphylococcus aureus* more abundant in fecal samples from babies born in Middlemore hospital (linear model, q = 0.016). Maternal antibiotic (Wilcoxon test, *p* = 0.013) and probiotic (*p* = 0.04) usage within the four-week period before sample collection was associated with a reduction in the alpha diversity of D10 samples. Infant probiotic intake explained 2.1% (PERMANOVA, *p* = 0.021) of the variance in the D10 microbial profile with increased *Lactobacillus* (linear model, q = 1.1 × 10^−10^) levels. At 4M, the microbiome of infants who were breastmilk fed had reduced alpha diversity when compared to non-breastmilk fed infants (Wilcoxon test, *p* < 0.05). Although causality cannot be inferred within our study, we conclude that in MLPT babies, maternal socioeconomic factors, as well as the perinatal medical environment and nutrition impact on the development of the newborn microbiome.

## Introduction

There are approximately 15 million babies born preterm each year (World Health Organization, 2017), with countries in Africa and South Asia accounting for more than 60% of all preterm births (World Health Organization, 2017). In New Zealand, between 2008 and 2017, 1.2% to 1.3% of all babies were born before 32 weeks of gestation, and an additional 5.9 to 6.3% of all babies were born moderate–late preterm (MLPT, between 32 and 36 weeks of gestation) (Ministry of Health, 2019), accounting for >80% of all preterm births (Ministry of Health, 2019). Preterm babies often require postnatal nutritional support to sustain growth following their early exposure to the extrauterine environment. However, there are no data from randomized trials to inform standardized nutritional practice for MLPT babies (Giannì et al., 2015; Harding et al., 2017; Alexander and Bloomfield, 2019), meaning that practice varies widely according to expert opinion.

Over the past decades, advances in molecular technology have led to the emergence of the microbiome and its importance in relationships between the environment and host characteristics (Blanton et al., 2016; Kamng’ona et al., 2019; Robertson et al., 2019). In preterm babies, alteration in the gut microbiome composition is hypothesized to be a determining factor leading to the development of necrotizing enterocolitis (NEC), a gut complication with high morbidity and mortality (Musemeche et al., 1986; Morowitz et al., 2010; Neu and Walker, 2011). A systematic review of 14 studies provided some support for this hypothesis, reporting that preterm infants diagnosed with NEC are characterized by an increased abundance of Proteobacteria and decreased abundance of Firmicutes and Bacteroidetes from 24 to 36 weeks corrected gestational age (Pammi et al., 2017).

Studies into the early life gut microbiota have been focused around extremely preterm, very preterm (Gregory et al., 2016; Arboleya et al., 2017), term, and healthy infants (Biasucci et al., 2010; Akagawa et al., 2019) or a mixture of term and preterm (Chernikova et al., 2018; Dahlgren et al., 2019; Fouhy et al., 2019). Feeding mode, chronological age, and birth weight have been reported to influence gut microbial composition in preterm infants <32 weeks of gestation over the first three months of life (Gregory et al., 2016; Cong et al., 2017). For example, very-low-birth-weight infants fed mother’s own breastmilk have been reported to have higher microbial alpha diversity, improved feed tolerance and better growth 4–6 weeks after birth compared to infants fed donor human milk (Ford et al., 2019). It has previously been shown that the microbiomes of MLPT babies that were fed mothers’ own breastmilk up to 15 days had similar alpha diversity, but significantly distinct beta diversity levels to formula fed MLPT babies (Wang et al., 2020). A meta-analysis of seven microbiome studies comparing the gut microbiome of exclusively and non-exclusively breastfed infants identified reduced alpha diversity in the former group (Ho et al., 2018).

There have been a number of studies that have focused on the acquisition and establishment of the infants’ gut microbiome from birth (Dahlgren et al., 2019; Shao et al., 2019; Tauchi et al., 2019; Li et al., 2020). However, it remains uncertain how the microbiome of MLPT babies develops across the early life window. In this study, we focused on identifying associations between early life dietary nutrition, perinatal medical environments, and socioeconomic factors, and the gut microbiome of MLPT babies. Anthropometric measurements were collected at birth and 4-months corrected age (4M). Fecal samples were collected at Day-10 (D10), and 4M and were used to investigate associations with the anthropometric and social data. Our results provide insights that will contribute to the long-term optimization of health outcomes for MLPT babies.

## Materials and Methods

### DIAMOND Trial Description and Ethics

The DIAMOND trial (Bloomfield et al., 2018), is a multi-center, factorial design, randomized, controlled clinical trial (Trials Registry: ACTRN12616001199404). Briefly, the DIAMOND trial enrols MLPT infants who have an intravenous line for clinical reasons and whose mothers intend to breastfeed to investigate the impact of current feeding strategies on feed tolerance, body composition, and developmental outcome (Bloomfield et al., 2018). Exclusion criteria are babies in whom a particular mode of nutrition is clinically indicated, or who have a congenital abnormality that is likely to affect growth, body composition, or neurodevelopmental outcome.

Ethical approval was obtained from the New Zealand Health and Disability Ethics Committee (number 16/NTA/90). Institutional approval for each site [Counties Manukau Health (Middlemore Hospital); Auckland District Health Board (Auckland City Hospital); and Waitemata District Health Board (North Shore and Waitakere Hospitals)] was obtained through local institutional review processes. Written, informed, consent was required from parents or legal guardians prior to enrolment. The DIAMOND trial is overseen by an independent data and safety monitoring committee.

### Probiotics

Use of prophylactic probiotics was undertaken according to each hospital’s policy. The probiotics that were given to babies during admission, if required, included: *Lactobacillus GG* (Dicoflor60 Dicofarm SpA), Infloran^®^ (SIT, Laboratorio Farmaceutico, Mede, Italy) (*Bifidobacterium bifidum* and *Lactobacillus acidophilus*) or Labinic™ Drops (Biofloratech Ltd, UK) (*Lactobacillus acidophilus*, *Bifidobacterium bifidum* and *Bifidobacterium infantis*).

### Sample Collection and Transportation

The fecal samples analyzed in this study were collected from babies enrolled in the DIAMOND trial between March 2017 and June 2019 from four hospitals in Auckland, New Zealand (*i.e.* Auckland City Hospital; Middlemore Hospital; North Shore Hospital, and Waitakere Hospital). Fecal samples were collected by the MLPT babies’ parents or nursing staff at two time points, day-10 (D10) (chronological age) and at 4-months corrected age (4M). Protocols for stool collection were standardized at both time points (**Data Sheets 2** and **3** for D10 and 4M respectively) to minimize the introduction of uncontrolled variables (Vogtmann et al., 2017).

For D10 samples, all fecal samples were frozen (−20°C) immediately after collection. Frozen samples were transported, on ice, to The Liggins Institute within five days of collection.

4M fecal samples were collected by parents/legal guardians following a detailed protocol that included illustrations from collection through to storage (**Data Sheet 3**). Parents/legal guardians were requested to freeze the collected fecal sample before transporting it (on ice) to the follow-up appointment.

### DNA Extraction

DNA was extracted from 200 mg of fecal matter per sample within 7 days of collection using the Allprep DNA/RNA Mini Kit (QIAGEN), using a modification of Giannoukos et al. (2012). The fat layer that floated on top of the supernatant, after cell lysis, was carefully removed to avoid clogging the extraction column. The quality and quantity of the extracted DNA were measured using a NanoPhotometer N60 (IMPLEN, Germany) prior to storage and Qubit (Invitrogen, US) prior to sequencing. Extracted DNA was stored at −80°C until use.

### Metadata Processing

*Socioeconomic status (SES)* was assigned according to the census-based New Zealand geography deprivation index (NZDep2013) using the parents’ self-reported postcode. NZDep index assigns a deprivation score ranging from 1 to 10 for each meshblock (a geographical unit that represent area where people live and containing a median of around 81 people) in New Zealand that applies to areas rather than individual people (Atkinson et al., 2014). SES was then categorized into three groups: higher SES (NZDep Index 1–3), moderate SES (NZDep Index 4–7), or lower SES (NZDep Index 8–10).

*Maternal education level* was classified as either university (*i.e*. bachelor, masters, or doctoral degree level) or no university (*i.e.* no education, primary, lower secondary (years 9–11), upper secondary (years 12–13), post-secondary non-tertiary course or short-cycle tertiary education).

*Gestational age* was classified into moderate (32^+0^ to 33^+6^ weeks gestation) or late gestation (34^+0^ to 35^+6^ weeks gestation).

*Early life intravenous nutrition* was classified as: intravenous nutrition (babies received both amino acid solution and dextrose), or only dextrose before stool collection at D10.

*Types of milk feeding on day-10* was classified as: breastmilk feeding (breastmilk only, or a combination of breastmilk and bovine-origin fortifier); formula only (term and/or preterm formula); or mixed feeding (a mix of breastmilk, formula, and/or bovine-origin fortifier in breastmilk).

*Types of milk feeding at 4-months corrected age* was categorized into three groups: breastmilk feeding; formula only; or mixed feeding (including weaning foods) based on self-reported information provided by parents at the 4M follow-up appointment.

Anthropometric measurements were expressed as the delta z-score which corrected for gestational age and sex (z-score, based on Fenton and World Health Organisation (WHO) growth charts) using the following formula:

$$delta_{z-score}=4\;month_{z-score}-birth_{z-score}$$

#### Amplicon Sequencing and Data Analysis

16S rRNA amplicon libraries were prepared using the Nextera XT kit (Illumina). The V3–V4 16S rRNA hypervariable region was amplified using the universal primers 341F (5′-CCTACGGGNGGCWGCAG-3′) and 805R (5′-GACTACHVGGGTATCTAATCC-3′). 16S rRNA amplicon sequencing was performed using an Illumina MiSeq sequencing platform (Auckland Genomics, School of Biological Sciences; The University of Auckland, New Zealand). Amplicon sequence information is available at the Sequence Read Archive (SRA) under BioProject Accession Number PRJNA645223.

Adapter trimming was performed using Cutadapt (Martin, 2011) and reads were fed into the DADA2 pipeline (version 1.13.3 (Callahan et al., 2016)) in R (version 3.5.0) for quality control, denoising, and sequence merging (including removal of PhiX reads and chimeric sequences). The resulting amplicon sequence variants (ASVs) were taxonomically annotated using the SILVA database (SSU release 132). Seven samples had fewer than 3,000 16S amplicon sequencing reads and were excluded from further analysis (**Supplementary Table S1**). The remainder of the samples had filtered sequence counts ranging from 6,098 to 74,597 reads (median reads = 33,026, **Supplementary Table S1**).

#### Metagenomics Sequencing and Initial Bioinformatics

Shotgun metagenomic libraries were generated using the NEBNext^®^ Ultra™ DNA Library Prep Kit (Illumina). Shotgun metagenomic sequencing (150 bp paired-end reads) was performed on a NovaSeq 6000 platform (Annoroad Gene Technology Beijing Co Ltd). All metagenome sequence information was deposited in the SRA (https://www.ncbi.nlm.nih.gov/sra) under the BioProject Accession Number PRJNA648487. KneadData (http://huttenhower.sph.harvard.edu/kneaddata) was used for quality control and to remove contaminant reads (*e.g.* human genome; **Data Sheet 1**, **Supplementary Figure 1**) from the metagenomic data. Our samples contained <10% human-aligned reads (**Supplementary Table S2**), consistent with current estimates for stool samples (Marotz et al., 2018). MetaPhlAn2 was used for taxonomic profiling (Truong et al., 2015). Metabolic pathway reconstruction was performed using the MetaCyc database and the HUMAnN2 pipeline (The HMP Unified Metabolic Analysis Network 2; (Franzosa et al., 2018)).

### Bioinformatic and Statistical Analyses

Statistical analyses were conducted using R (version 3.5.0 and 3.6.1). Microbial alpha diversity was measured using Shannon’s diversity index. Permutational multivariate analysis of variance (PERMANOVA; adonis function in vegan R package, version 2.5-6 (Oksanen et al., 2019), 10,000 permutations) was used to quantify the contributions of covariates to the observed variance in microbial beta diversities. Associations between individual microbial taxa, that were present in ≥10% of the samples and other variables were tested using Multivariate Association with Linear Models (MaAsLin2) (Morgan et al., 2012). Wilcoxon unpaired (R package rstatix version 0.3.0) (Kassambara, 2019) and Kruskal–Wallis tests (R Core Team, 2019) were used to compare two and ≥two independent groups, respectively. The Chi-square test of independence and Fisher’s exact test (R Core Team, 2019) were used to determine the association between two categorical variables. All reported p-values in this study were corrected for multiple testing using the Benjamini–Hochberg procedure (p. adjust function) (R Core Team, 2019) (Benjamini & Hochberg, 1995; Chen et al., 2017). By convention, FDR corrected p-values from MaAsLin2 were reported as q-values.

## Results

### Study Cohort

**Figure 1:**
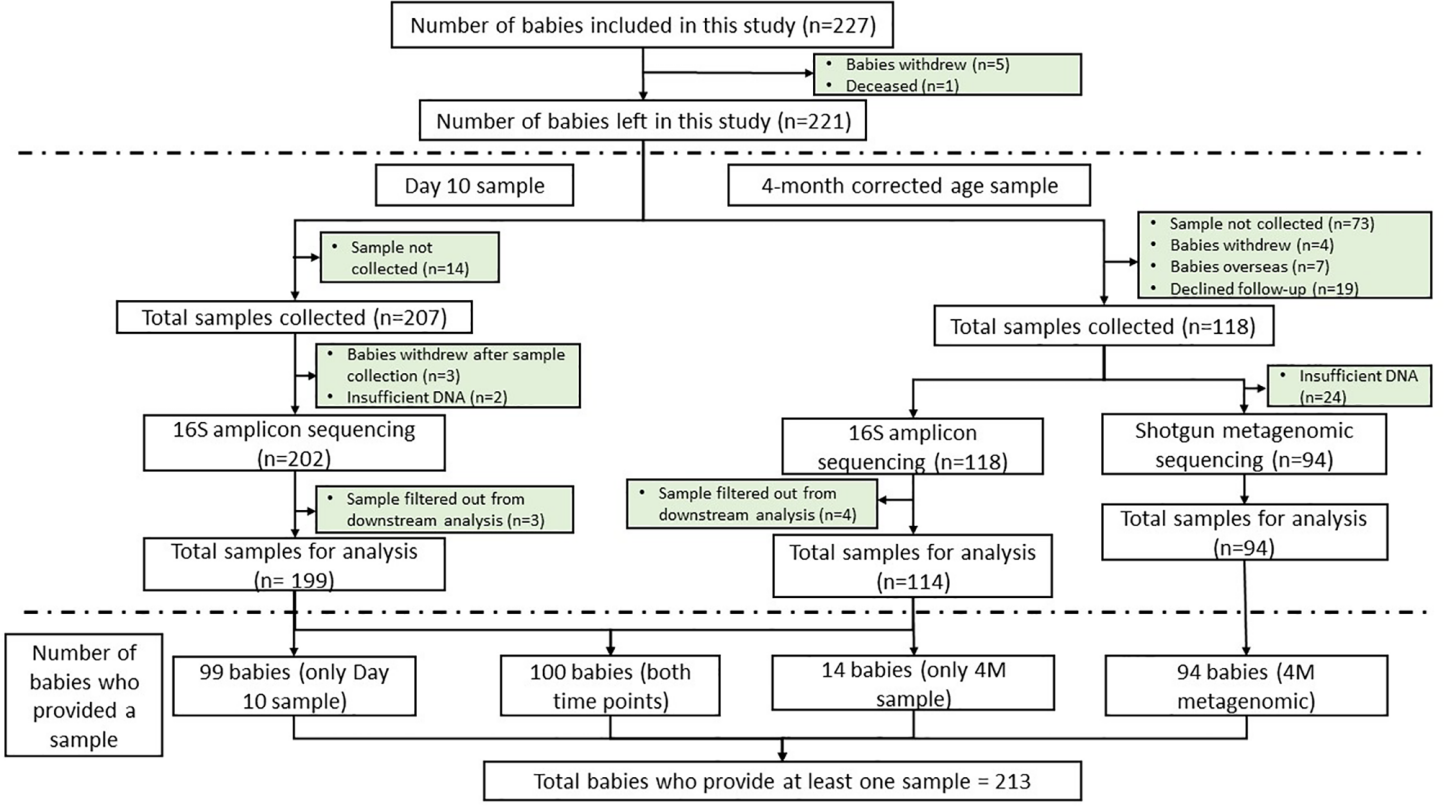
Consort diagram showing the number of babies included in this study (*n* = 227), fecal samples collected (*n* = 325) and the number of babies excluded from the study at each time point. The consort diagram includes the numbers of samples that were available for downstream analysis and the equivalent number of babies that provided sample at each time point. Reasons for exclusion are stated.

In total, 227 babies were included in this study. Five babies withdrew from the study and one died before sample collection (**Figure 1**). Of the 325 stool samples that were collected from 221 babies (n = 207 D10 and n = 118 4M), 320 underwent 16S amplicon sequencing, and 313 samples were analyzed in the downstream analysis. Specifically, 100 babies provided stool samples at both time points, 99 babies provided only D10 sample, 14 babies provided only 4M sample (**Table 1** and **Figure 1**). Three babies did not provide a fecal sample at either time point. Proportional analysis performed on the demographic data identified a significantly different sex distribution (test of proportions, *p* = 0.026) and Cesarean section rates (*p* = 2.44 × 10^−8^) between the time points but not gestation age. The difference in Cesarean section rates was also significant within sex strata (D10: *p* = 7.86 × 10^−5^ and 4M: *p* = 0.0001), but no significant difference was found in gestational age within the sex strata.

**Table 1:** Subject cohort demographic and clinical data.

Baby	
Birth weight (g)	2106 ± 426
Birth length (cm)	45 ± 3
Birth head circumference (cm)	31 ± 2
Weight at 4-months corrected age (g)	6,638 ± 926
Length at 4-months corrected age (cm)	64 ± 3
Head circumference at 4-months corrected age (cm)	42 ± 1
Neonatal intensive care unit (NICU) stay (days)	22 ± 11
Hospital stay (days)	24 ± 11
**Mother**	
Maternal age (years)	32 ± 6
**Samples for 16S amplicon sequencing**	
**Sample collection (days)** Day-10 4-months corrected age	10 ± 1 157 ± 14
**Sex (Male/Female)** Day-10 4-months corrected age	112/87 66/48
**Mode of delivery (Vaginal/Caesarean section)** Day-10 4-months corrected age	70/129 35/79
**Gestational age (Moderate/Late)** Day-10 4-months corrected age	112/87 57/57
**Samples for shotgun metagenomics**	
**Sex (Male/Female)**	58/36
**Mode of delivery (Vaginal/Cesarean section)**	28/66
**Gestational age (Moderate/Late)**	48/46

Gestational age: moderate preterm (32 + ^0^ to 33 + ^6^ weeks of gestational age) and late preterm (34 + ^0^ to 35 + ^6^ weeks of gestational age). Data are mean ± standard deviation. For birth weight, n = 213; birth head circumference, n = 213; birth length, n = 210; 4M-weight, n = 174; 4M-length, n = 172, 4M-head circumference, n = 174.

A subset of 94 4M fecal samples (36 female and 58 male), which contained sufficient DNA (≥155 ng), was sent for metagenomic shotgun sequencing (**Table 1** and **Figure 1**). As infants’ growth differs by season (Gelander et al., 1994; Bozzola and Meazza, 2012) we ensured a representation of samples from individuals in each season. Specifically, among the 36 female babies, six babies were born in winter, 10 each in spring, summer, and autumn. Of the male babies: 23 were born in winter, 12 in spring, nine in summer, and 14 in autumn.

### Longitudinal Changes in the Gut Microbiome

Longitudinal analysis of the fecal microbiome using 16S amplicon data from infants (*n* = 100) who were sampled at both D10 and 4M time points revealed that 5.7% of the variance in fecal microbial profile is explained by longitudinal changes (PERMANOVA, *p* = 0.001). The fecal microbial alpha diversity varied according to time point of sampling (Wilcoxon paired test, *p* = 5.64 × 10^−10^) with the 4M fecal samples showing significantly greater alpha diversity when compared to D10 (**Data Sheet 1**, **Supplementary Figure 2**). This observed increase in fecal microbial diversity is consistent with our current understanding of the development of the early life microbiome (Avershina et al., 2014; Fouhy et al., 2019). The abundance of all four major gut phyla—Firmicutes, Actinobacteria, Proteobacteria, and Bacteroidetes—changed significantly between the two time points (linear model, q < 0.1). Within these phyla, eight taxa from the phylum Firmicutes (*i.e. Veillonellaceae, Ruminococcaceae, Erysipelotrichaceae, Peptostreptococcaceae*, and *Lactobacillaceae*), five taxa from Actinobacteria (*i.e. Bifidobacteriaceae, Eggerthellaceae, Coriobacteriaceae, Actinomycetaceae*, and *Atopobiaceae*), one taxon from Proteobacteria (*i.e. Enterobacteriaceae*) and one more taxon from Bacteroidetes (*i.e. Bacteroidaceae*) were more abundant in 4M fecal samples when compared to the D10 samples. By contrast, one taxon each from Firmicutes (*i.e. Staphylococcaceae*), Actinobacteria (*i.e. Corynebacteriaceae*) and Proteobacteria (*i.e. Pasteurellaceae*) were less abundant in 4M samples (**Supplementary Table S4**—MaAsLin2 longitudinal analysis, doi: 10.17608/k6.auckland.12793772).

### Socioeconomic Factors Were Associated With Infants’ Fecal Microbiome Diversity Up to 4-Months Corrected Age

**Figure 2:**
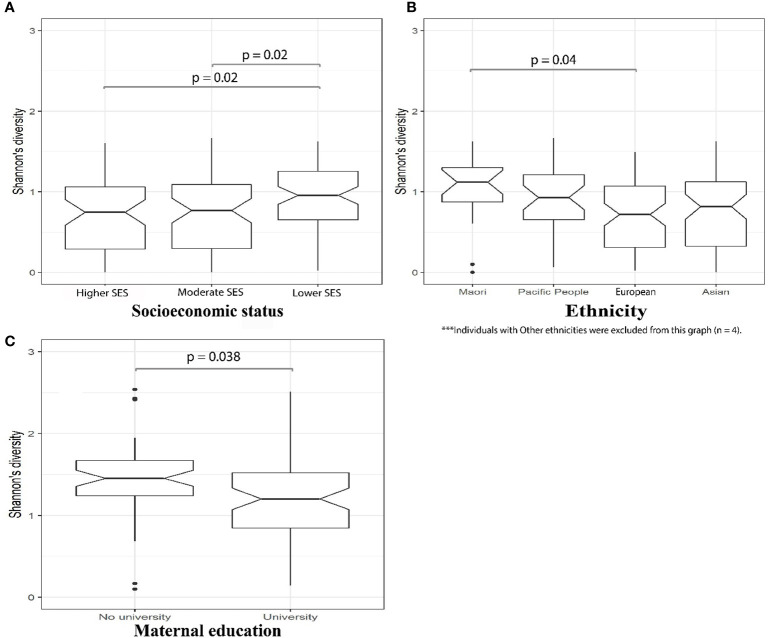
16S amplicon sequencing identified significantly (*p* = 0.02, Wilcoxon) greater microbial alpha diversity in D10 fecal samples from MLPT babies whose families lived in lower SES areas **(A)**; D10 fecal samples from MLPT babies born to mothers self-reporting as Māori exhibited significantly higher alpha diversity compared to babies from mothers self-reporting as European (*p* = 0.04, Wilcoxon) **(B)**; and 4M fecal samples from babies whose mothers hold a university degree had significantly (*p* = 0.038, Wilcoxon) lower microbial alpha diversity when compared to babies whose mothers do not have a university degree **(C)**.

In our MLPT cohort, we observed a correlation between early life microbial alpha diversity (D10, Shannon diversity index), SES, and self-reported maternal ethnicity. We observed a significantly higher microbial diversity in babies whose families lived in the lower SES areas (NZDep Index 8–10) when compared to the other two groups (**Figure 2A**, Wilcoxon test, *p* = 0.021). Notably, there was higher abundance for members of the *Bifidobacterium* (phylum Actinobacteria; linear model, q = 0.02) and *Megasphaera* (phylum Firmicutes; linear model, q = 0.031) genera in babies from lower SES group compared to the higher SES group at D10 and 4M, respectively (**Supplementary Table S4**—MaAsLin2—D10 (16S data) & 4M (16S data), doi: 10.17608/k6.auckland.12793772).

Mothers who self-reported as Māori were over-represented in lower SES areas (75%), compared to the other ethnicities in our MLPT cohort (**Supplementary Table S3**). Therefore, we tested for an association between self-reported maternal ethnicity and the infants’ gut microbial diversity in D10 fecal samples. We observed that the alpha diversity of the D10 fecal microbiome of babies born to mothers self-reporting as Māori was the highest and significantly different to that from babies born to mothers self-reporting as European (Wilcoxon test, *p* = 0.04; **Figure 2B**). Linear modeling revealed that the genus *Rothia* (phylum Actinobacteria) (q = 0.061) was reduced at 4M in babies born to mothers self-reporting as Māori when compared to those born to mothers self-reporting as Asian. The genus *Staphylococcus* (phylum Firmicutes) was less abundant in fecal samples obtained at 4M from MLPT babies born to mothers self-reporting as Māori (q = 0.011) and Pacifica (q = 0.031) when compared to MLPT babies from mothers self-reporting as Asian.

We detected a negative correlation between infants’ microbial alpha diversity at 4M with maternal education level (Wilcoxon test, *p* = 0.038). Specifically, babies whose mothers held a university degree had a reduced fecal microbial alpha diversity compared to babies whose mothers did not have a university degree (**Figure 2C**). Chi-square test of independence and Fisher’s exact test identified a significant correlation between maternal education with both SES and maternal self-reported ethnicity at both time points (D10: SES, Chi-square test, *p* = 0.0002; ethnicity, Fisher’s exact test, *p* = 0.00002; 4M: SES, Chi-square test, *p* = 0.036; ethnicity, Fisher’s exact test, *p* = 6.01 × 10^−7^). Specifically, this indicates the inter-relatedness of SES, maternal self-reported ethnicity and maternal education at both D10 and 4M.

### Breastmilk Feeding Was Associated With a Reduction in Microbial Diversity at 4 Months

Intravenous nutrition is an essential component of the medical care for preterm infants before they can tolerate full enteral feeds. No associations between the fecal microbiome and early life intravenous nutrition (a combination of amino acids and dextrose solution or only dextrose) were identified at D10 (PERMANOVA, R^2^ = 0.002, *p* = 0.91) or 4M fecal samples (PERMANOVA, R^2^ = 0.013, *p* = 0.581) by 16S amplicon data. Similarly, PERMANOVA analysis on the types of milk feeding in the early life from birth to D10 (*i.e.* breastmilk only, formula only, or mixed feeding) with fecal microbial composition in D10 (R^2^ = 0.020, *p* = 0.172) or 4M (R^2^ = 0.023, *p* = 0.581) samples did not identify any associations.

**Figure 3:**
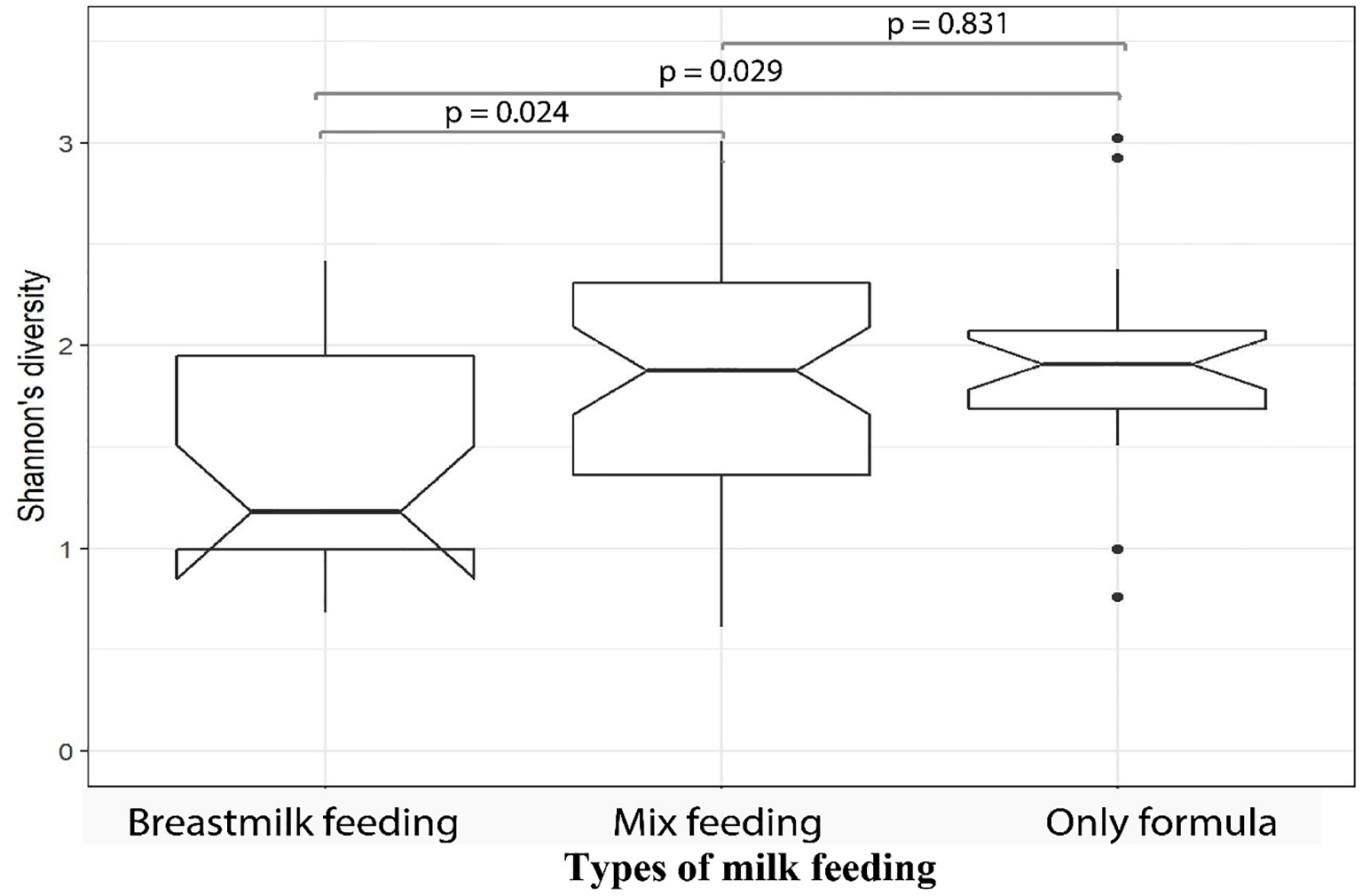
Fecal samples from MLPT infants, at four-months corrected age, who were breastmilk fed exhibited lower microbial alpha diversities when compared to mixed fed (*p* = 0.024, Wilcoxon) and formula fed (*p* = 0.029, Wilcoxon) infants. The notch inversion indicates that the lower confidence level is less than the first quartile.

We then investigated the microbial alpha diversity at 4M in infants who were subject to different types of milk feeding (*i.e.* breastmilk, formula, or mixed). We observed that the types of milk feeding were associated with changes in diversity (Kruskal–Wallis test, *p* = 0.032). Specifically, significantly lower alpha diversity was observed in infants who received breastmilk when compared to mix fed babies (Wilcoxon test, *p* = 0.039), using 16S amplicon data. This observation was supported by metagenomics data at 4M where lower microbial alpha diversity was observed in 4M fecal samples collected from breastmilk fed infants when compared to infants who received only formula (Wilcoxon test, *p* = 0.029), or those who were mix fed (Wilcoxon test, *p* = 0.024; **Figure 3**). PERMANOVA analysis of 16S amplicon data also revealed that types of milk feeding explained 6.5% (*p* = 0.001) of the microbial taxonomic variation that was observed at 4M. Specifically, the genus *Megasphaera* from the Firmicutes phylum was more abundant in 4M fecal samples from breastmilk fed babies when compared to babies that were fed only formula (linear model, q = 0.051; **Supplementary Table S4**—MaAsLin2—4M (16S data), doi: 10.17608/k6.auckland.12793772).

### Maternal and Infant Medical Factors Impact the Infants’ Gut Microbial Diversity

**Figure 4:**
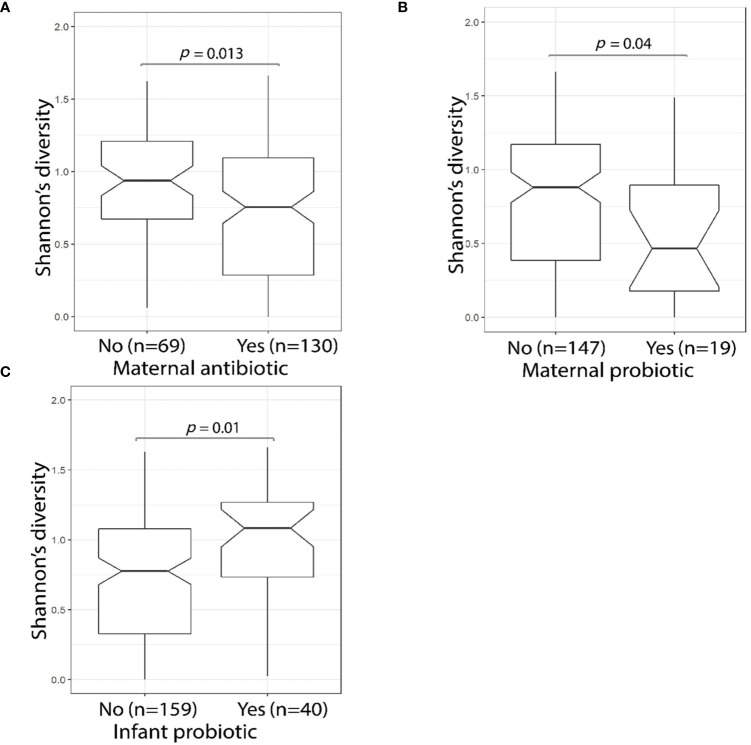
MLPT fecal microbiome alpha diversity was affected by maternal antibiotic and probiotic usage. The Shannon’s diversity index was calculated using 16S amplicon sequencing data obtained from MLPT infants’ D10 fecal samples. Mother’s antibiotic usage within the four weeks preceding the child’s D10 sample collection was associated (*p* = 0.013, Wilcoxon) with a decrease in MLPT infants’ fecal alpha diversity **(A)**; a significant reduction in microbial alpha diversity was also observed in infants whose mothers used probiotics (*p* = 0.04, Wilcoxon) in the four weeks preceding D10 sample collection **(B)**; and infants who received probiotics during admission had higher fecal microbial alpha diversity when compared to their counterparts who did not receive probiotics (*p* = 0.01, Wilcoxon) **(C)**.

Maternal use of antibiotics or probiotics within the four-week period before the D10 fecal sample collection was associated with a significant reduction (Wilcoxon test, *p* = 0.013 and *p* = 0.040, respectively) in MLPT infant fecal sample microbial alpha diversity (**Figures 4A, B** **)**. By contrast, D10 fecal samples of infants who themselves received probiotics during their admission had significantly greater alpha diversity levels when compared to infants who did not (Wilcoxon test, *p* = 0.01, **Figure 4C**). PERMANOVA analysis confirmed that infant probiotic usage explained 2.1% (*p* = 0.021) of the variance in the D10 fecal microbial profile. *Lactobacillus*, a commonly used probiotic, was more abundant in the fecal sample collected from MLPT babies that received probiotics (n = 40) when compared to babies that did not receive probiotics (n = 159, linear model, q = 1.1 × 10^−10^, **Supplementary Table S4**—MaAsLin2—D10 (16S data), doi: 10.17608/k6.auckland.12793772). No association was observed in the fecal microbial alpha diversity from babies that received probiotics (Wilcoxon test, *p* = 0.118, 10/113) within one month immediately preceding the 4-month follow-up appointment.

Infant antibiotic use during neonatal admission had no effect on fecal microbial alpha diversity at D10 (Wilcoxon test, *p* = 0.50, n = 102/199). Similarly, we observed no significant difference in alpha diversity levels in 4M fecal samples from the small number of babies (n = 5/113) that received antibiotics within the month immediately preceding the 4-month follow-up appointment (Wilcoxon test, *p* = 0.118). The levels of genus *Clostridium* were higher in 4M fecal samples from babies who received antibiotics within the month immediately preceding the 4-month follow-up appointment (linear model, q = 0.098, **Supplementary Table S4**—MaAsLin2—4M (16S data), doi: 10.17608/k6.auckland.12793772). However, the significance of these findings is limited by the numbers of individuals who received antibiotics (4.4%, n = 5/113, after excluding samples with missing data) and probiotics (8.8%, n = 10/113, after excluding samples with missing data) within the month immediately preceding the 4-month follow-up appointment.

We used HUMAnN2 to analyze the 4M shotgun metagenomics data to identify the gene complements of the samples. We identified a significant increase (linear model, q = 0.018) in the counts of genes involved in the allantoin degradation pathway (MetaCyc identifier PWY0-41) (**Supplementary Table S4**—HUMAnN2—4M (metagenomic data), doi: 10.17608/k6.auckland.12793772) in fecal samples from babies that received antibiotics (n = 4/93, after excluding samples with missing data) within the month immediately preceding the 4-month follow-up appointment when compared to babies that did not received antibiotics (n = 89/93, after excluding samples with missing data). The small number of babies who received antibiotics means this observation is underpowered.

### The Gut Microbiome Composition Correlated With Growth Velocity

Evidence indicates there is an association between fecal microbial alpha diversity and the early growth rate of babies (Blanton et al., 2016; Gehrig et al., 2019; Vatanen et al., 2019). We examined the relationship between the fecal microbiome and change in MLPT weight, head circumference and length over the first four months of life. We computed the change (delta) z-score between birth and 4-months corrected age for the anthropometric growth measurements we collected. The change in weight and head circumference z-scores was associated with the beta diversity of the microbial metagenomic profiles from fecal samples collected at 4M in female (PERMANOVA, weight: R^2^ = 0.050, *p* = 0.042; head circumference: R^2^ = 0.051, *p* = 0.042), but not male, MLPT babies (PERMANOVA, weight: R^2^ = 0.019, *p* = 0.566; head circumference: R^2^ = 0.019, *p* = 0.566; **Supplementary Table S5**, doi: 10.17608/k6.auckland.12793811). No correlation was found between the delta z-score of length and the 4M fecal microbial profile in either male or female MLPT babies (male: R^2^ = 0.009, *p* = 0.938; female: R^2^ = 0.021, *p* = 0.735).

### Gestational Age, Mode of Delivery, and Plurality Influence the Establishment of the Gut Microbiota

We performed a PERMANOVA analysis of Bray–Curtis dissimilarity to identify other factors that are associated with changes in the MLPT fecal microbiome composition at D10 and 4M. Gestational age (*i.e.* moderate and late preterm) explained 1.2% of the variation in D10 fecal microbial beta diversity (*p* = 0.043). Mode of delivery contributed 2.1% of the variance (*p* = 0.002) within D10 fecal samples, with *Bacteroides* being more abundant in babies born vaginally (linear model, q = 0.002, **Supplementary Table S4**—MaAsLin2—D10 (16S data), doi: 10.17608/k6.auckland.12793772). However, neither gestational age (R^2^ = 0.014, *p =* 0.173) nor mode of delivery (R^2^ = 0.008, *p =* 0.452) were identified as contributing to the variation observed in the MLPT fecal microbial profile at 4M. Notably, plurality explained 2.6% of the variance (*p* = 0.034) in the microbial profile for samples collected at 4M, with a higher abundance of *Eggerthella lenta* observed in twins (n = 31/94) when compared to singletons (n = 63/94, linear model, q = 0.109, **Supplementary Table S4**—MaAsLin2—4M (metagenomic data), doi: 10.17608/k6.auckland.12793772).

### The Impact of Hospital Environment and Maternal Associated Factors on the Establishment of the MLPT Fecal Gut Microbiota at 4M

The hospital environment was associated with changes to the microbial profile (Bray–Curtis) at 4M, where hospital of birth explained 5.2% of the observed variance (PERMANOVA, *p* = 0.038) in the shotgun metagenomic data. For example, *Staphylococcus aureus* was more abundant in the 4M microbiome of babies born at Middlemore hospital (linear model, q = 0.016) when compared to babies born at Auckland City hospital. By contrast, analyses of the 16S rRNA amplicon data did not identify an association between hospital of birth and infants D10 (PERMANOVA, R^2 =^ 0.024, *p =*0.084) and 4M fecal microbial profile (PERMANOVA, R^2^ = 0.038, *p =* 0.150). Similarly, analyses of the 4M data using Fisher’s exact test did not identify differences in either early life antibiotic (*p* = 0.725) or probiotic usage (*p* = 0.866) between the birth hospitals. However, analyses of the 4M 16S rRNA amplicon data did identify an association between length of time that the MLPT baby stayed in hospital and microbial profile (Bray–Curtis), which explained 2.4% of the variance (PERMANOVA, *p* = 0.034) (**Supplementary Table S5**, doi: 10.17608/k6.auckland.12793811).

There was no association identified between the fecal microbial beta diversity at D10 (PERMANOVA, R^2^ = 0.003, *p* = 0.970) or 4M (PERMANOVA, R^2^ = 0.014, *p* = 0.320, **Supplementary Table S5**, doi: 10.17608/k6.auckland.12793811) and maternal age. Despite this, the level of the genus *Sutterella* (phylum Proteobacteria) in 4M fecal samples was inversely associated with maternal age (linear model, q = 0.051, **Supplementary Table S4**—MaAsLin2—4M (16S data), doi: 10.17608/k6.auckland.12793772). None of the other maternal factors we tested (*i.e.* stress and depression level) correlated with the alpha or beta diversity of the infants’ gut microbiota at either time point (**Supplementary Table S5**, doi: 10.17608/k6.auckland.12793811).

## Discussion

*Bifidobacterium* is a common early life commensal in healthy-full term babies (Nagpal et al., 2017). Therefore, it was notable that *Bifidobacterium* species were at low abundance in fecal samples from our MLPT cohort at D10. The low abundance of *Bifidobacterium* observed in our D10 cohort could be explained by a number of factors. First, a majority (*i.e.* 63.8%) of the babies in our cohort were delivered by Cesarean section and *Bifidobacterium* has been reported to be in low abundance in term-born Cesarean section babies (Biasucci et al., 2010; Tannock et al., 2013). Secondly, *Bifidobacterium* is a genus that is well-known to be associated with breastfeeding (Tannock et al., 2013; Stewart et al., 2018). In MLPT babies, breastfeeding takes longer to be established. This delay may have contributed to the reduction observed. Thirdly, it might be related to the consequences of prematurity (*e.g.* composition of breastmilk and maturity of the infant gut) as in 45 preterm breastfed babies *Bifidobacterium* abundance has been reported to be associated with corrected postmenstrual age, increasing gradually after 30 weeks of postmenstrual age (Korpela et al., 2018). Therefore, we conclude that the combined effect of being born preterm and being born by Caesarean section delivery contributed to the observed low abundance of *Bifidobacterium* in MLPT children. With respect to a long-term effect, it is known that the numbers of *Bifidobacteria* peak after birth and progressively decrease to a stable number in adulthood (Arboleya et al., 2016). We speculate that the low abundance we observed in early life might not affect the level of *Bifidobacterium* that is attained in adulthood due to the compositional changes of *Bifidobacterium* species with respect to ageing (Gavini et al., 2001; Kato et al., 2017). However, this requires confirmation through long-term longitudinal studies of the MLPT population.

We observed higher alpha diversity in the D10 fecal microbiomes of babies whose mothers had lower socioeconomic status (SES) and babies born to mothers who self-reported as being of Māori ethnicity. Superficially, this would appear to be consistent with observations that associate socioeconomic disparity and diet with gut microbial composition and richness (Miller et al., 2016; Bowyer et al., 2019). However, the effect of SES on the gut microbiota remains controversial (Chong et al., 2015; Miller et al., 2016; Bowyer et al., 2019; Gschwendtner et al., 2019). In our study, this was further confounded by collinearities between ethnicity and SES rendering it difficult to untangle SES effects from a few other factors in our study. Further work needs to be undertaken to provide greater understanding of the effects of SES on the microbiome.

The differences observed at day 10 fecal microbiomes dissipated over time such that there was no association between the infants’ gut microbiota composition and maternal ethnicity or SES in samples collected from our MLPT cohort at four months. This could be explained by a portion of maternal microbes that were vertically transferred to infants. However, we offer alternative explanations for this lack of differences at 4M. First, we observed a significant negative correlation between maternal education levels with infants’ microbial alpha diversity at 4M, and maternal education levels are significantly colinear with both maternal self-reported ethnicity and SES in our MLPT cohort, similar to previous reports (Easton, 2013). Secondly, the common commensals present within the mothers’ microbiomes might not be retained following early life vertical transmission due to a requirement for specific nutrients. Thirdly, the acquisition of these founder bacteria in early life might have been subject to a “dilution effect” as a result of nutrients present in the breastmilk or formula being bioavailable to bacteria that grow to outnumber these founder organisms.

No association was identified between infants’ D10 fecal microbiome composition and hospital of birth. By contrast, an association between infants’ 4M fecal microbiome composition with hospital of birth and length of hospital stay were observed. It is notable that D10 fecal sample was collected 10 days after birth yet, on average, babies in our cohort spent 22 days in the hospital. Therefore, it remains possible that the hospital of birth does impact the microbiome but that the establishment of this effect, by cross-transmission from other infants, or inoculation from hospital workers or environment is established over a longer period than the initial sampling time and thus not seen in the fecal sample collected at D10.

The influence of environmental factors on the gut microbiota composition has been shown to surpass that of the host genetics in two large cohorts of healthy adults [n= 1,046 (Rothschild et al., 2018) and n= 858 (Scepanovic et al., 2019)]. Support for the impact of environmental effects is further substantiated by Koo et al. (2019) who showed that twins separated for decades shared fewer bacteria strains compared to twins who cohabitated for a long time (Koo et al., 2019). Our findings demonstrated that plurality (*i.e.* singleton versus twins) was associated with 4M fecal microbial beta diversity. Notably, we did not observe a difference in D10 fecal sample microbial profile. Other than the vertical transfer of microbiome from mothers to infants, twins shared a common environment after hospital discharge, which makes the environmental effect more prominent. We argue that the combined effect of interactions between genetic variation and environmental factors surfaced later in life when the infants are no longer sharing a common environment.

The increased abundance of Firmicutes in our longitudinal analysis of fecal microbiome development in MLPT infants from D10 to 4M was consistent with previous observations (Bäckhed et al., 2015) as a hallmark of the maturation of the gut microbiome (Stewart et al., 2018). Gestational age at birth and mode of delivery were observed to associated with D10 fecal microbial beta diversity. This observation is consistent with an earlier study that identified a significant effect of gestational age at birth on the microbiome composition (Fouhy et al., 2019). Our observation that this effect was not clearly identifiable at 4M indicates it is weak and transient and agrees with other studies that have indicated the microbiomes of preterm and term babies converge later in life (Jayasinghe et al., 2020). Similarly, delivery mode has a maximum impact on the infants’ establishing gut microbiome during the first week of life (Reyman et al., 2019; Shao et al., 2019), consistent with our observation that the effect of delivery mode dissipated at 4M in the MLPT cohort. However, the demographic differences between the time points and potential sample collection biases between hospital (D10) and home sampling (4M) may confound these or any other findings of this study.

We saw an increase in *Staphylococcus* abundance in 4M fecal samples only in children born to mothers who self-reported as Asian. High levels of *Staphylococcus* were associated with vaginal birth and infants born early (Korpela et al., 2018). We contend that the increase we observed in the Asian subgroup was because a larger proportion of births were vaginal in this group (31/77, 40.3%). However, a recent study of 554 South African women has identified *Staphylococcus, Rothia*, and *Gemella* as among the most abundant genera present in human breast milk (Ojo-Okunola et al., 2019). Therefore, it remains possible that the increased abundance of *Staphylococcus, Gemella*, and *Rothia* that was associated with ethnicity at D10 and 4M was due to different types of milk feeding. For example, more Māori and Pacifica mothers practised breastmilk feeding compared to Asian mothers at D10. However, the opposite was observed at 4M. In other words, types of milk feeding, together with maternal lifestyle and cultural practice, contribute to the process of establishing the gut microbial composition during early life.

Feeding regime (*i.e.* breastfed and formula-fed) has been previously identified as significantly affecting the infants’ gut microbiome composition (Azad et al., 2013; Praveen et al., 2015). In our MLPT cohort, we observed an increase in the abundance of *Megasphaera* spp. in breastmilk fed babies at 4M. This agrees with previous observations in a full-term Danish cohort where breastfeeding duration is positively associated with *Megasphaera* levels (Laursen et al., 2016). Lactate is more abundant in exclusively breastfed infants (Bridgman et al., 2017). The lactate utilization properties of *Megasphaera* are suggested to reduce lactate toxicity and to generate short-chain fatty acids (SCFAs) (Shetty et al., 2013). The low alpha diversity we observed in fecal samples from 4M breastmilk fed infants corroborates the findings of a previous meta-analysis on exclusively breastfed infants (Ho et al., 2018) of seven microbiome studies and a recent study on the exclusivity breastmilk feeding of infants at 3 months of age (Fehr et al., 2020). Therefore, we contend that HMOs and other compounds within breastmilk select for a specific, albeit low diversity microbiome.

The administration of prophylactic antibiotics to mothers undergoing Cesarean section is a common practice that is recommended by the World Health Organisation (Smaill and Grivell, 2014; World Health Organization, 2015; Liu et al., 2016). Maternal intrapartum antibiotic prophylaxis (IAP) was associated with changes in infants’ fecal microbial beta diversity (Shao et al., 2019) and reduced infants’ fecal microbial alpha diversity over the first three months of life (Nogacka et al., 2017). Our observation that maternal antibiotic and probiotic usage correlated with the infants’ D10 gut microbial alpha diversities is consistent with a mechanism that affects vertical microbial transmission from mothers to infants after birth. Given the association of feeding regime and birth mode with diversity, it remains possible that this transmission occurs through breastmilk and mode of delivery. As such, treatment of maternal medical conditions during the perinatal period might impact on the infants’ microbial profile during the most vulnerable postnatal period where breastmilk is the primary food source. However, the fact that the association with antibiotic usage was not seen at four-months corrected age indicates that it is not the dominant effect and is substitute by other environmental and feeding practices.

Healthy infants have predictable patterns of weight gain and growth (*i.e.* length and head circumference). Growth parameters are thus often used to reflect the overall health and nutritional status of an infant. However, infant growth does differ by seasons (greater in spring to summer compared to autumn to winter) (Gelander et al., 1994; Bozzola and Meazza, 2012) and is best calculated over at least a 6 month period. The ability of our study to address growth by seasons was limited by the fact that our growth records were: 1) only taken over a 4-month period; 2) only for MLPT infants; and 3) unevenly distributed over the seasons for the male babies. Despite these limitations, our results suggested a correlation of the gut microbiota composition with the growth velocity (delta z-score) in weight and head circumference of female babies. Kamng’ona et al. (2019) have identified an association between the gut microbiome and babies’ weight gain but not in length and head circumference in a mixed genders cohort (Kamng’ona et al., 2019). The composition of breast milk differed when mothers gave birth to boy and girl (Galante et al., 2018). As both male and female babies in our cohort were not treated differently in terms of nutrition, it remains possible that these gender differences led to different nutritional needs that contributed to our observations in female but not male MLPT babies.

## Conclusion

Our study demonstrates the complexity of factors (*i.e.* maternal socioeconomic factors, perinatal medical environment, gestational age and delivery mode) that impact upon gut microbiota acquisition and establishment in MLPT infants over the first 10 days after birth. These factors are largely substituted by infants’ immediate environment and types of milk feeding, which exert the dominant effects on the microbiome at 4-months corrected age. Finally, we highlighted the need to analyze male and female microbiomes separately, when looking at associations with growth.

## Data Availability Statement

The datasets presented in this study can be found in online repositories. The names of the repository/repositories and accession number(s) can be found in the article/**Supplementary Material**.

## Ethics Statement

The studies involving human participants were reviewed and approved by the New Zealand Health and Disability Ethics Committee (number 16/NTA/90). Written informed consent to participate in this study was provided by the participants’ legal guardian/next of kin.

## Author Contributions

Conceptualization, FB, TA, and JO’S. Data curation, CC, TV, FB, and JO’S. Formal analysis, CC and TV. Funding acquisition, FB, JO’S, and TA. Investigation, CC, TV, FB, and JO’S. Methodology, CC, TV, FB, TA, and JO’S. Supervision, TV, FB, and JO’S. Visualization, CC, TV, FB, and JO’S. Writing—original draft, CC. Writing—review and editing, CC, TV, FB, and JO’S. All authors contributed to the article and approved the submitted version.

## Funding

This work was supported by the Health Research Council of New Zealand (ID#: 16/605) and Counties Manukau Health.

## Conflict of Interest

The authors declare that the research was conducted in the absence of any commercial or financial relationships that could be construed as a potential conflict of interest.
