# Supplementary material for: Factors Associated With the Microbiome in Moderate–Late Preterm Babies: A Cohort Study From the DIAMOND Randomized Controlled Trial
Source: Front Cell Infect Microbiol. 2021 Mar 1;11:595323. doi: 10.3389/fcimb.2021.595323 (PMC7958882; doi:10.3389/fcimb.2021.595323)
Supplement: Supplementary file 1 [file DataSheet_1.docx]

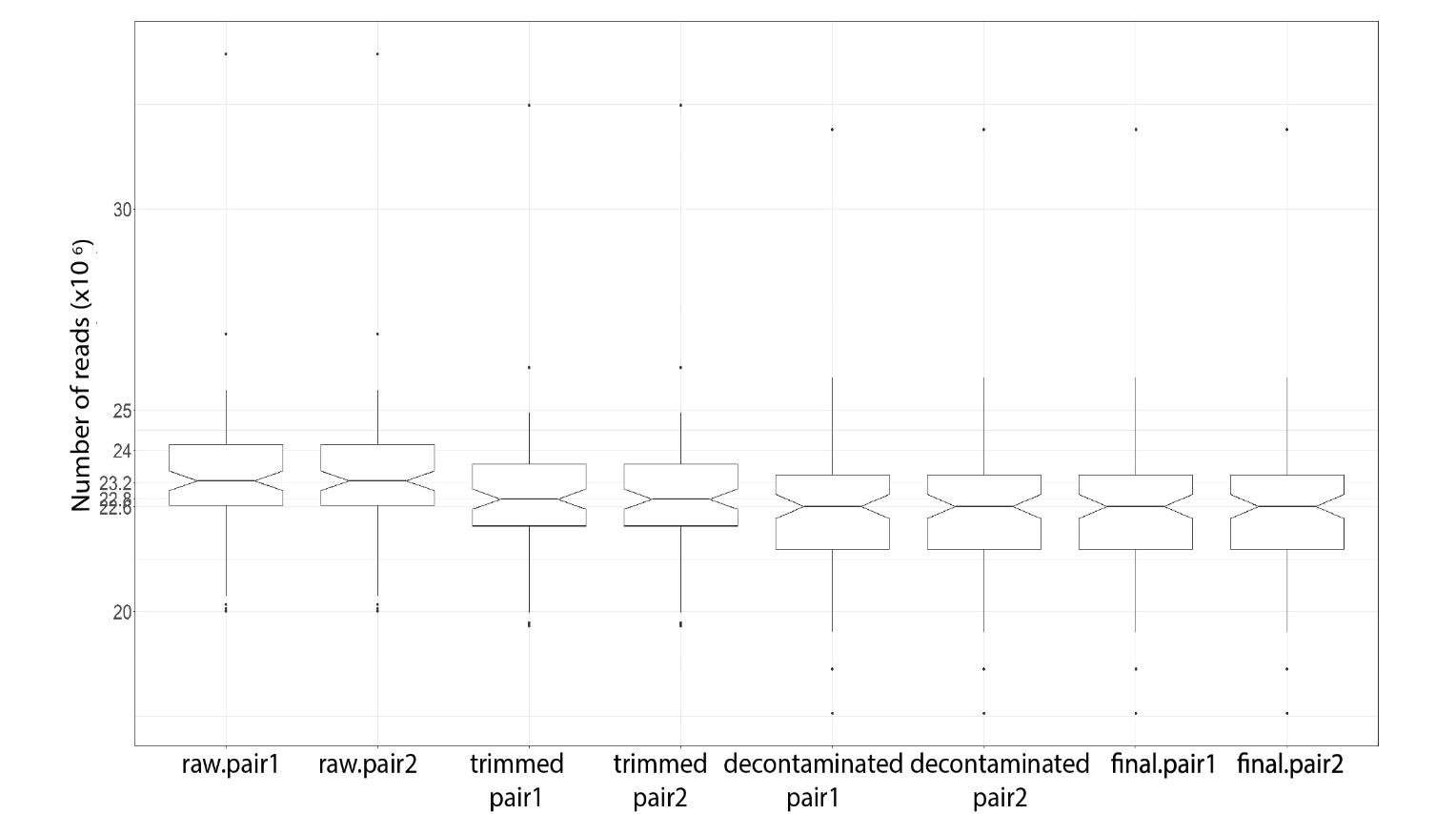


Supplementary Figure 1: Box and whisker plot of the number of shotgun metagenomic reads (x 10^6^) remaining after each quality control step using kneadData. Boxes indicate the interquartile range (IQR), the notch region shows the 95% confidence interval for the median. Whiskers represent the distribution within 1.5 times the IQR. Points outside of 1.5 x IQR are shown as outliers.


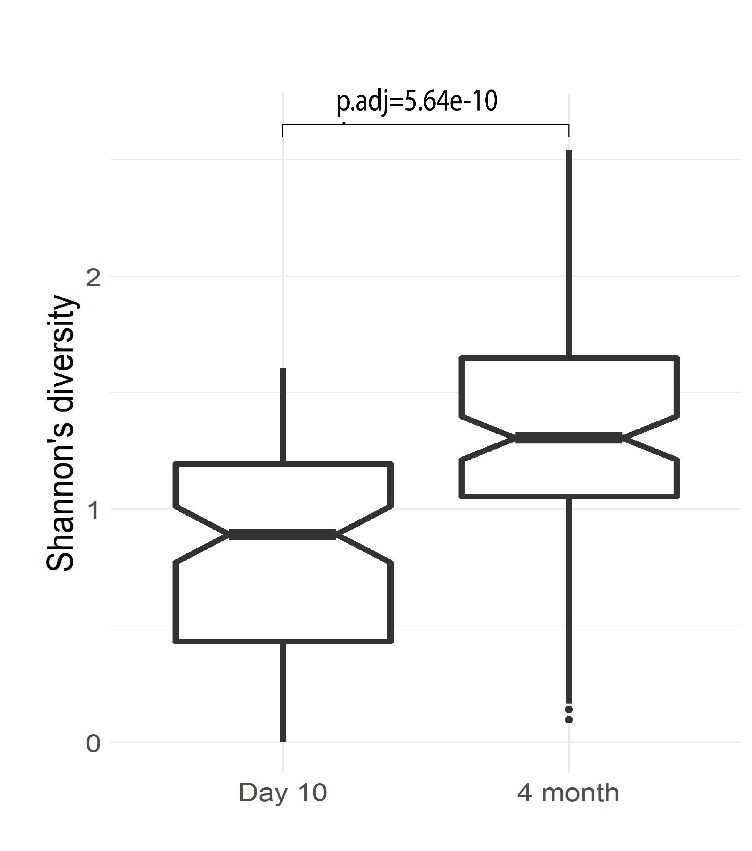


Supplementary Figure 2: The microbial alpha diversity, measured by Shannon’s diversity index, increased significantly from day 10 to 4-month corrected age (p = 5.64x10^-10^).
