# Supplementary material for: Factors Associated With the Microbiome in Moderate–Late Preterm Babies: A Cohort Study From the DIAMOND Randomized Controlled Trial
Source: Front Cell Infect Microbiol. 2021 Mar 1;11:595323. doi: 10.3389/fcimb.2021.595323 (PMC7958882; doi:10.3389/fcimb.2021.595323)
Supplement: Supplementary file 2 [file DataSheet_2.docx]

**Day 10 – Stool sample instructions**

A stool sample will be collected from all babies enrolled in the DIAMOND trial at 10 days of age (or the next bowel motion if not open on day 10):

1. Two stool samples will be collected from the same dirty nappy within 15 minutes of passage (or at the time of the nearest cares).
2.
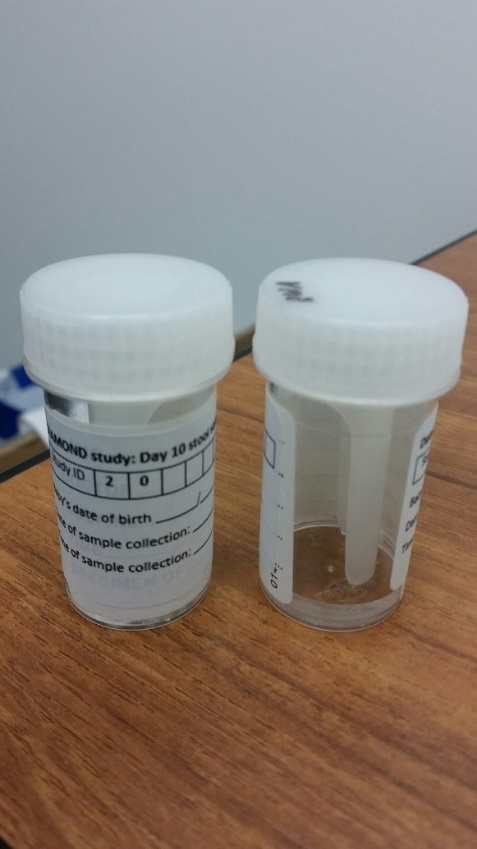
 The first sample will be collected using the RNAlater® white faeces cup lid (white top with RNA in black pen on top), scrape 1 scoop directly from stool in the nappy, tightly screw on cap and shake vigorously 10 times to ensure sample well mixed with solution.
3.
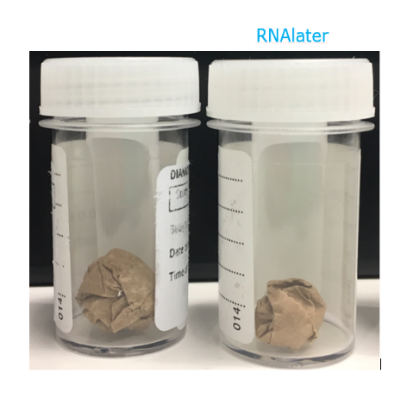
The second sample will be collected using the white faeces cup lid, scrape the remaining faeces (up to ½ of the tube) from the same dirty nappy as the RNAlater® sample and tightly screw lid back on.
4. Standard amount of stool collected is show in the picture.
5. Fill in the label on both samples be sure to correctly include study ID number, date and time of sample.
6. Place both samples into the same biohazard bag and place immediately into the DIAMOND freezer (-20^o^ C):
   1. At Auckland Neonatal Unit this is located in the blood gas room
   2. At Middlemore Neonatal Unit this is located in the sluice room on level 2
   3. At North Shore Hospital this is located in the sluice room
   4. At Waitakere Hospital this is located in the sluice room
7. The samples will need to transport to the Liggins Institute lab with 1 – 2 days of collection on cold chain transport this will be organized by the research team.
