# Supplementary material for: Factors Associated With the Microbiome in Moderate–Late Preterm Babies: A Cohort Study From the DIAMOND Randomized Controlled Trial
Source: Front Cell Infect Microbiol. 2021 Mar 1;11:595323. doi: 10.3389/fcimb.2021.595323 (PMC7958882; doi:10.3389/fcimb.2021.595323)

4 month Follow up – Instructions for Stool (Poo) Sample Collection

A poo sample will be collected from all babies enrolled in the DIAMOND trial at 4 month corrected age to look at the bugs that live in your baby's tummy.

Please collect two poo samples from the same dirty nappy <u>on the day before or the morning</u> that you and your baby are due to come back for the four months DIAMOND Trial visit.

Please follow the instructions below…

<table>
<tr><th colspan="3">Equipment</th></tr>
<tr><td colspan="2">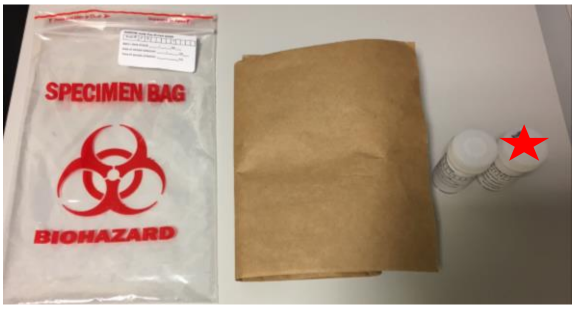
</td><td>In your preparation kit you will find a:
• Plastic 'specimen' bag
• Brown paper bag
• Two collection containers
! *The container with a star on the lid has liquid inside.* !</td></tr>
<tr><th colspan="3">What to do</th></tr>
<tr><td colspan="3">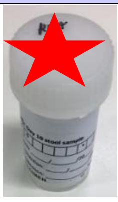
Use the collection container with a **star on top**</td></tr>
<tr><td>1</td><td>**Open the lid**
! Please **do not get rid of the liquid** in this container. This is supposed to be in there.</td><td>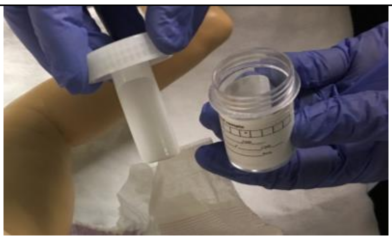</td></tr>
<tr><td>2</td><td>**Collect the sample**
There is a scoop attached to the lid.
Scrape one scoop of poo from the nappy.
Put the scoop with poo in inside the container.
Screw the lid back on tight.</td><td>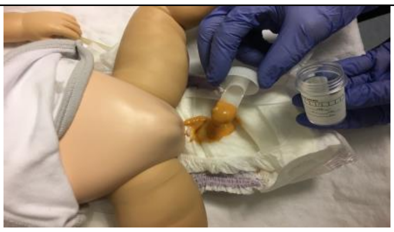
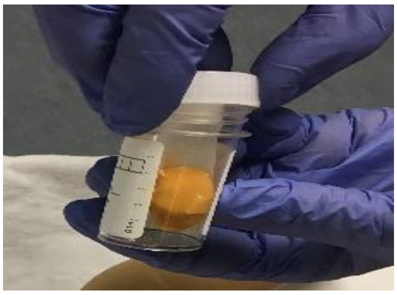</td></tr>
<tr><td>3</td><td>**Mix**
Shake the container so that the poo *mixes really well* with the liquid inside the container.</td><td>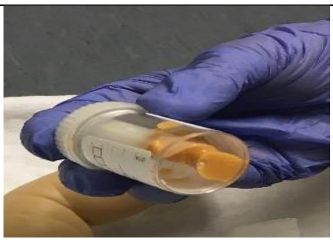</td></tr>
</table>

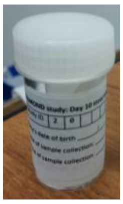

Using the other collection container…

1 **Collect the sample**

Place a scoop of poo into this container.

Do not fill more than half full.

Screw the lid back on tightly.

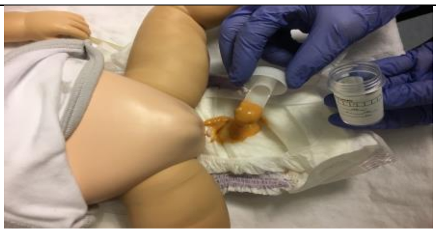

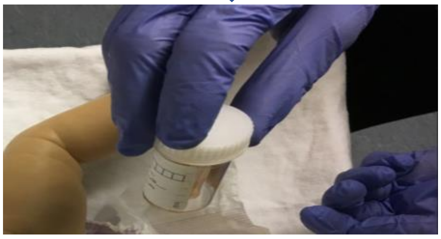

Storing

Please write the **DATE** and **TIME** the poo was collected on:

- the label of both containers, and
- the sticker on the plastic bag.

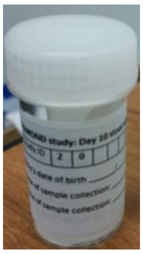

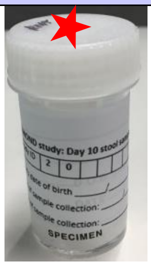

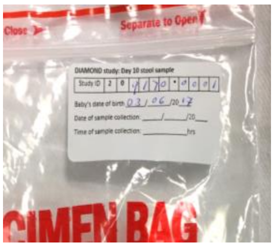

Put the containers into the plastic bag.

Put the plastic bag inside the paper bag.

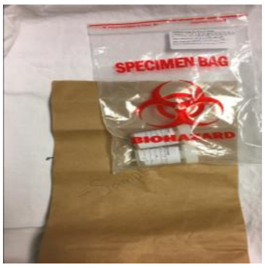

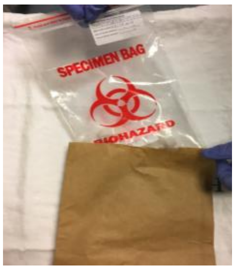

Please place this in your freezer ASAP.

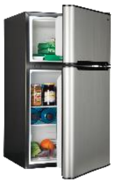

On the day of your appointment with us…

Please bring this bag with you on the day of your 4 month follow-up appointment, on ice if possible.

Thank you!

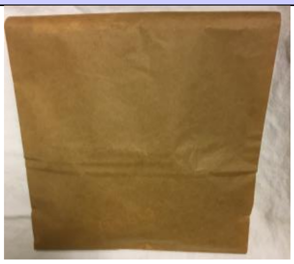

Supplement: Supplementary file 3 [file DataSheet_3.pdf]
